# Three-Dimensional Reconstruction from a Single RGB Image Using Deep Learning: A Review

**DOI:** 10.3390/jimaging8090225

**Published:** 2022-08-23

**Authors:** Muhammad Saif Ullah Khan, Alain Pagani, Marcus Liwicki, Didier Stricker, Muhammad Zeshan Afzal

**Affiliations:** 1 Department of Computer Science, Technical University of Kaiserslautern, 67663 Kaiserslautern, Germany; 2 German Research Institute for Artificial Intelligence (DFKI), 67663 Kaiserslautern, Germany; 3 Department of Computer Science, Luleå University of Technology, 971 87 Luleå, Sweden; 4 Mindgarage, Technical University of Kaiserslautern, 67663 Kaiserslautern, Germany

**Keywords:** deep learning, 3D reconstruction, convolutional neural networks, textureless surfaces

## Abstract

Performing 3D reconstruction from a single 2D input is a challenging problem that is trending in literature. Until recently, it was an ill-posed optimization problem, but with the advent of learning-based methods, the performance of 3D reconstruction has also significantly improved. Infinitely many different 3D objects can be projected onto the same 2D plane, which makes the reconstruction task very difficult. It is even more difficult for objects with complex deformations or no textures. This paper serves as a review of recent literature on 3D reconstruction from a single view, with a focus on deep learning methods from 2018 to 2021. Due to the lack of standard datasets or 3D shape representation methods, it is hard to compare all reviewed methods directly. However, this paper reviews different approaches for reconstructing 3D shapes as depth maps, surface normals, point clouds, and meshes; along with various loss functions and metrics used to train and evaluate these methods.

## 1. Introduction

Three-dimensional reconstruction is the task of inferring the geometric structure of a scene from a set of two-dimensional images. Given one or more 2D views of a scene, we want to know the 3D shape and position in space of all the objects in the scene. This can be seen as mapping the 2D points in the image space to 3D points in real-world space, where for each 2D point (x,y) in the image, we want to recover the corresponding 3D point $\left(\bar{x},\bar{y},\bar{z}\right)$ in world coordinates, where $\bar{z}$ is the distance of the point from the camera.

There are two main ways to reconstruct a 3D scene: monocular reconstruction from a single image, or multi-view reconstruction from multiple images taken from different perspectives. The goal of both tasks is to infer the 3D geometry of the scene, but monocular reconstruction can be more difficult because you only have one view of the scene. Multi-view reconstruction is easier because you get more information about the hidden faces of objects from different angles, making it easier to infer their shape and position.

The literature also distinguishes between the reconstruction of a whole scene as opposed to the reconstruction of a single object. A scene is usually made up of a set of objects, and scene reconstruction involves reconstructing not only the geometry of all the objects in the scene but also their relative positions. Reconstruction of a whole scene is generally harder than reconstruction of a single object because it involves dealing with more complex shadows and occlusions. An image can be a projection of infinitely many different shapes, which makes correctly reconstructing the 3D shape from a single image very hard. Reconstruction of the non-visible faces of the object is challenging in particular as the input image often provides no information about their shape. Bautista et al. [1] showed that many existing monocular 3D reconstruction networks learn shape priors over training object categories to solve this problem. This makes it difficult for these networks to generalize to unseen object categories or scenes. Tatarchenko et al. [2] demonstrated that single-view 3D reconstruction networks do not reason about the 3D geometry of the object from visual shape cues, but rather rely on object recognition to perform the reconstruction.

The reconstruction task is also made more difficult when there are no textures in the scene. Without any distinctive features, it becomes harder to infer the 3D shape of an object from a 2D image. This makes it difficult to create a complete and accurate 3D model of the scene.

Three-dimensional reconstruction from visual data is a long-standing computer vision problem with real-world applications in fields such as robotic surgery, autonomous vehicles, and virtual and augmented reality. In recent years, deep learning has replaced the traditional computer vision algorithms for the problem of 3D reconstruction with promising results. Deep learning networks have been shown to be more robust to noise and variations in input data than traditional methods. Additionally, they are able to learn complex features from data without any human intervention. This makes them well suited for tasks such as 3D reconstruction where there is a lot of variability in input images.

In this paper, we review different deep learning-based methods proposed for the task of 3D reconstruction from a single view. We only focus on methods of reconstructing individual objects. Our main contributions include:

- An overview of the neural networks proposed for monocular 3D object reconstruction in the last five years, including:
  - Bednarik et al. [3] and Patch-Net [4] for reconstruction of depth and normal maps using a real dataset of textureless surfaces;
  - HDM-Net [5] and IsMo-GAN [6], which reconstruct 3D point clouds from a synthetic dataset of textured surfaces;
  - Pixel2Mesh [7], Salvi et al. [8], and Yuan et al. [9] for reconstruction of mesh-based models using a subset of the ShapeNet [10].
- A summary of the major 3D datasets that are used by the discussed neural networks.
- A description of common metrics used to evaluate the 3D reconstruction algorithms.
- A comparison of the performance of these methods using different evaluation metrics.

The remainder of the paper is organized as follows. In Section 2, we present an overview of related literature. Section 3 defines various methods of representing 3D data, such as depth maps, normal maps, point clouds, 3D meshes, and voxels. In Section 4, we introduce the major 3D datasets used by the networks reviewed in this paper. Then, we discuss different deep learning methods proposed recently for 3D reconstruction in Section 5. Section 6 defines the evaluation metrics used by these networks and lists the results of various experiments conducted by these methods on different datasets. We further discuss the experiment results in Section 7 before concluding our findings and providing direction for future work in Section 8.

## 2. Related Work

**Figure 1:**
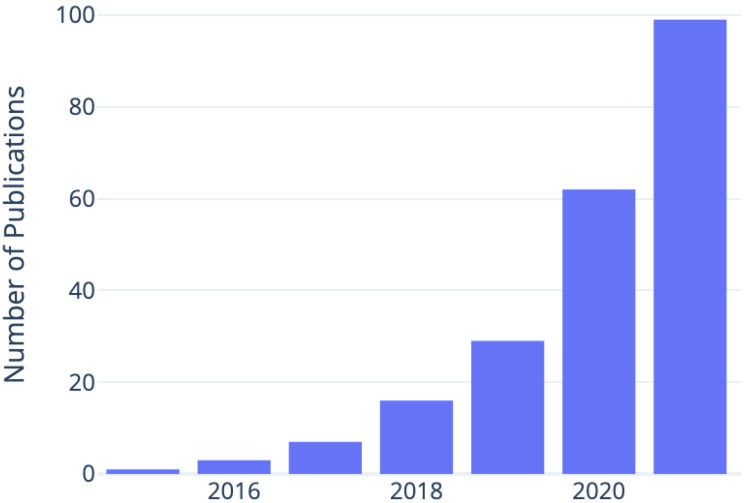
Interest in using deep learning-based methods for 3D reconstruction is reflected in the number of publications on ScienceDirect matching the keywords “3d reconstruction” AND “deep learning”, which have been exponentially growing since 2015.

The problem of 3D reconstruction from visual data has been well-studied in computer vision literature, but reconstructing 3D geometry from images remained an ill-posed problem before 2015 when researchers started using convolutional neural networks for this task. Figure 1 shows the trend of related publications since then. In this section, we shed some light on existing surveys that have previously reviewed the 3D reconstruction methods in the literature.

Zollhöfer et al. [11] published a report in 2018 on the state-of-the-art in monocular 3D reconstruction of faces. The authors mainly focus on optimization-based algorithms for facial reconstruction, but also briefly mention the emerging trend of using learning-based techniques for this task. They conclude that they “expect to see heavy use of techniques based on deep learning in the future”. In 2019, Yuniarti and Suciati [12] formally defined 3D reconstruction as a learning problem and showed an exponentially growing interest in 3D reconstruction among the deep learning community. This paper talks about the different ways of representing shapes in 3D, such as parametric models, meshes, and point clouds, lists the 3D datasets available at that time, and summarizes various deep learning methods for 3D reconstruction. Han et al. [13] published a more extensive review of single and multi-view 3D reconstruction later that year. This work also distinguishes between the reconstruction of scenes and reconstruction of objects in isolation, and reviews techniques for both.

A large body of work in this area focuses solely on producing depth maps from images, which represent a partial representation of 3D geometry. In this context, Laga [14] extensively surveyed more than 100 key contributions using learning-based approaches for recovering depth maps from RGB images. More reviews published in the following years show the shift in trend from using plain CNNs to recurrent neural networks (RNNs), residual networks, and generative adversarial networks (GANs) for 3D reconstruction with encouraging results [15,16]. Fu et al. [17] also published a review of single-view 3D reconstruction methods, focusing only on objects in isolation. They cover networks proposed between 2016 and 2019 in their review.

## 3. Representing Shape in 3D

There are many different ways to represent the 3D shape of a scene. The depth and normal maps can be used to represent the partial geometry of the scene which is limited to the surfaces of the objects directly facing the camera. For a more complete representation, point clouds, meshes, and voxels can be used. The 3D reconstruction networks can be trained to reconstruct 3D scenes from 2D images in different ways. In this section, we briefly introduce different ways of representing 3D data.

### 3.1. Depth Map

For each pixel in an image, a depth map provides the distance from the camera to the corresponding point in space. This gives a single-channel image of the same size as the input image, with the corresponding depth value, *z* at each (x,y) position. The absolute depth values are sometimes mapped to the range [0, 255] and, together with a normal RGB image, the depth map is given as the fourth channel of the so-called RGB-D images with points closer to the camera appearing darker and the points further away appearing brighter.

As depth maps are created from a single viewpoint, they represent a very sparse 3D geometry of the scene containing only points directly in the line of sight of the camera. They say nothing about the occluded planes, nor do they say anything about the 3D orientation of different faces of an object in the scene. For this reason, RGB-D images are sometimes called 2.5D because they cannot represent a complete 3D topology on their own. They are a partial surface model, with only the shape of the front face of the surface represented.

### 3.2. Normal Map

A normal vector or a “normal” to a surface is a three-dimensional vector perpendicular to the surface at a given point. Analogous to depth maps, normal maps provide normals for each pixel in an image. This means that we can tell the 3D orientation of the surface at any given point in space that is visible in this image.

When RGB-D datasets are combined with normal maps, we can extract both the distance and orientation of every point in a scene from a single viewpoint. However, since we are only seeing the scene from one perspective, information about the hidden surfaces of the objects in the scene cannot be completely represented. Like depth maps, normal maps also represent a partial surface model.

### 3.3. Point Cloud

A point cloud is a set of 3D points in space. Theoretically, they are able to exactly represent a complete 3D scene by storing the position of every point in space. Depending on how many and which points are present in the point cloud, it can be both a solid and a surface model of the scene. However, due to limited computational memory, it is often necessary to downsample them to reduce the size of the dataset. This can be done by removing the points which are very close to each other or which are not needed to understand the visible shape of the 3D surfaces.

Point clouds can be extremely useful for representing 3D shapes, but they can also be difficult to work with. Sometimes it is necessary to convert them to a mesh in order to get a more accurate representation of the object.

### 3.4. 3D Mesh

A mesh is a collection of 3D points (or vertices) that have been connected together with edges to form the surfaces (or faces) of 3D objects. Vertices are connected in a way that the faces are made up of many polygons adjacent to each other. Usually, these polygons are triangles, and the meshes are called “triangulated meshes”. Meshes can be used to represent the surface models of a 3D scene as “wireframes”.

### 3.5. Voxel

A voxel is a 3D equivalent of a pixel. Voxel-based models represent objects as a collection of cubes (or voxels) stacked in space like a 3D grid. They represent a discretized, solid model of the scene. Accuracy of the 3D model depends on the size of the voxels making up the objects. The bigger the voxels, the more “pixelated” the surfaces of the objects appear.

Like meshes, voxel grids can also be generated directly from point clouds where several adjoining points are all approximated to a single voxel (or a cube) in space. This process is called voxelization and is one way of downsampling the point clouds.

## 4. Datasets

In this section, we introduce the datasets used by the networks discussed later in Section 5. These datasets contain RGB images of different objects and their 3D shape in one of the representations introduced above. Table 1 provides a summary of these datasets.

### 4.1. Real Textureless Surfaces Data

**Figure 2:**
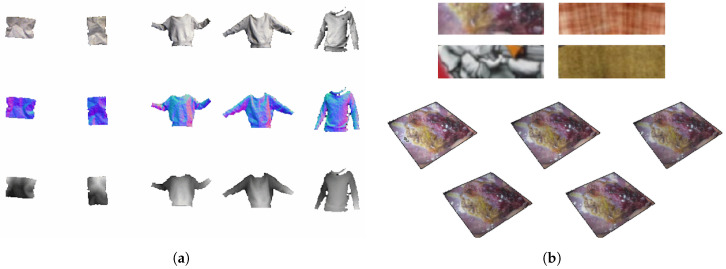
Examples of images in the datasets of Bednarik et al. [3] and Golyanik et al. [5], which are used to evaluate some of the networks in this paper. (**a**) The textureless surfaces dataset [3] contains RGB images and corresponding normal and depth maps for 5 different real objects. (**b**) The synthetic point cloud dataset of Golyanik et al. [5] has a deforming thin plate rendered with 4 different textures under 5 different illuminations.

This RGB-D dataset of deformable textureless surfaces from [3] consists of 26,445 RGB images, along with depth maps and normal maps for each image. These RGB images and depth maps were collected using a Microsoft Kinect for Xbox One device. Normal maps were computed by differentiating the depth maps. The dataset contains five different types of objects: tshirt, hoody, sweater, a rectangular sheet of cloth, and a crumpled piece of paper. The objects have no texture or colors on them. Figure 2a shows some samples from this dataset.

The tshirt, hoody, and sweater were worn by a person who made random motions to simulate realistic creases. The sheet of cloth was fixed to a bar on the wall and manually deformed, and the piece of paper was crumpled by hand to create different depths. Different combinations of four light sources were used to create lighting variations across different recording sequences. This included three fixed lights in front of the objects on the right, left, and center, and one moving dynamic light in the room. Table 2 summarizes the number of samples of each kind of object.

### 4.2. Synthetic 3D Point Cloud Data

Golyanik et al. [5] generated a synthetic 3D dataset in point cloud representation. Using Blender [19], they created a 3D scene with a thin plate undergoing various isometric non-linear deformations. Four kinds of textures (endoscopy, graffiti, clothes, and carpet) were mapped onto the deformed 3D model, which was then illuminated in various settings using five different light sources. The scene was viewed from five separate cameras at different angles. In this way, a total of 4648 states were generated. Each state is represented with $73^{2}$ 3D points sampled on a regular grid at rest and a consistent topology across states. For each state, there is also a corresponding rendered 2D image viewing the object from one of the cameras. Figure 2b shows some samples from this dataset.

### 4.3. ShapeNet

ShapeNet [10] is a large-scale dataset containing richly annotated 3D CAD models organized according to the WordNet [20] hierarchy. It contains over 300 M models, 220 M of which are classified into 3135 WordNet symsets [21]. ShapeNet is made up of many smaller subsets. A major subset is called ShapeNetCore, which contains manually verified single clean 3D models. It has two versions, v1 and v2, covering 55 and 57 categories respectively. Processed meshes and annotations of these models can be downloaded online from the ShapeNet website [22].

### 4.4. R2N2 Dataset

Choy et al. [18] took a subset of the ShapeNetCore v1 dataset containing 50,000 models and 13 categories including plane, bench, cabinet, car, chair, monitor, lamp, speaker, firearm, sofa, table, phone, and watercraft. For each object, the R2N2 dataset also makes available its own 24 renderings of the models from different viewpoints in a 137×137 resolution, and the 3D models themselves as meshes, point clouds and voxels.

## 5. Networks

This section introduces some of the recent methods proposed for reconstructing 3D surfaces from a single 2D image. These are summarized in Table 3.

### 5.1. Bednarik et al.

**Figure 3:**
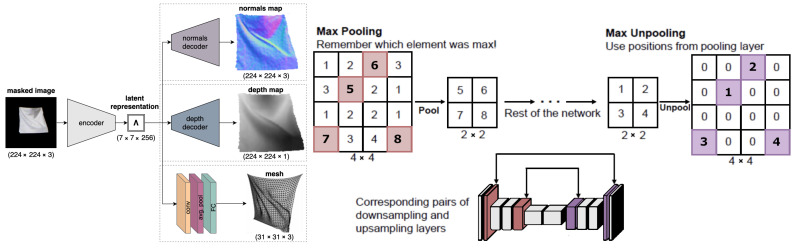
The textureless surface reconstruction network [3] (**left**) consists of an encoder Λ that takes a masked image $I_{m}^{n}$ as input and outputs a latent representation Λ. This is followed by three parallel decoders $\Phi_{N},\Phi_{D},$ and $\Phi_{C}$ that use Λ for reconstructing the normal map, depth map, and a 3D mesh respectively. The indices of all maxpool operations in the encoder are saved when downsampling (**right**). These indices are later used for non-linear upsampling in corresponding decoder layers.

Bednarik et al. [3] introduced a general framework for reconstructing the 3D shape of textureless surfaces with an encoder–decoder architecture. Using a single RGB image, they reconstruct the normal maps, depth maps, and triangulated meshes for the objects in the images. Figure 3 shows an overview of their architecture. This network has an encoder connected to three separate decoders, one each for reconstructing the normal map, depth map, and the triangulated mesh. The encoder takes an RGB image of size 224×224×3 and creates a latent representation of size 7×7×256. This encoding is fed to the three decoders.

The architecture of the encoder and the depth and normal decoders is based on SegNet [23]. The encoder has the same layers as VGG-16 [24] except for the fully convolutional layers. However, in contrast to VGG-16, the output channels at the convolutional blocks are 32,64,128,256,264 respectively. As the normal maps and depth maps have the same spatial size as the input image, the normal and depth decoders are symmetric to the encoder with both having the same architecture except the number of channels at the final output layer; the normal decoder has three channels and depth decoder has one channel. Like SegNet, pooling indices at the max pooling layers in the encoder are saved, and used in the normal and depth decoders to perform non-linear upsampling. For the mesh decoder, a smaller network with a single convolutional layer followed by average pooling and a fully connected layer is used.

The depth decoder is trained by minimizing the absolute difference between the predicted and ground-truth depth values of the foreground, giving the loss function

(1) $$L_{D}=\frac{1}{N}\sum_{n=1}^{N}\frac{\sum_{i}\left|D_{i}^{n}-\Phi_{D}{\left(\Lambda (I_{m}^{n})\right)}_{i}\right|B_{i}^{n}}{\sum_{i}B_{i}^{n}},$$

where $D^{n}$ is the ground-truth depth map and $\Phi_{D}$ is the depth decoder, which takes the encoder output on the masked input image $\Lambda (I_{m}^{n})$ and returns the predicted depth map. The absolute difference is only calculated for the foreground pixels, i.e., where the foreground mask $B^{n}$ has the value 1, and the sum of absolute differences is averaged over all the foreground pixels.

To train the normal decoder, the angular distance between the predicted and ground-truth normal vectors and the length of the predicted normal vectors are both optimized using the loss function

(2) $$L_{N}=\frac{1}{N}\sum_{n=1}^{N}\frac{\sum_{i}\left(\kappa L_{a}\left(N_{i}^{n},{\bar{N}}_{i}^{n}\right)+L_{l}\left({\bar{N}}_{i}^{n}\right)\right)B_{i}^{n}}{\sum_{i}B_{i}^{n}}$$

with

(3) $$\begin{array}{cc} L_{a}\left(N_{i}^{n},{\bar{N}}_{i}^{n}\right) & =\arccos\left(\frac{N_{i}^{n}{\bar{N}}_{i}^{n}}{∥N_{i}^{n}∥∥{\bar{N}}_{i}^{n}∥+\epsilon }\right)\frac{1}{\pi }, \end{array}$$

(4) $$\begin{array}{cc} L_{l}\left({\bar{N}}_{i}^{n}\right) & ={\left(∥{\bar{N}}_{i}^{n}∥-1\right)}^{2} \end{array}$$

where $L_{a}$ is the angular distance calculated as the arccos of the cosine similarity between the predicted and ground-truth normal vectors, $L_{l}$ is the term that prefers unit normal vectors, and κ is a hyperparameter that sets the relative influence of the two terms. Furthermore, $N^{n}$ is the ground-truth normal map, and the ${\bar{N}}^{n}=\Phi_{N}\left(\Lambda (I_{m}^{n})\right)$ is the predicted normal map. As with depth loss, the normal loss is only calculated for foreground pixels.

Finally, for the triangulated mesh prediction, the mesh decoder optimizes the mean squared error between predicted and ground-truth vertex coordinates. That is,

(5) $$L_{C}=\frac{1}{N}\sum_{n=1}^{N}\frac{1}{V}\sum_{i=1}^{V}{∥v_{i}^{n}-\Phi_{C}\left(\Lambda (I_{m}^{n})\right)∥}^{2}$$

As all three decoders take input from the same encoder with the same latent representation, they can be trained either jointly or separately. When trained jointly, the authors of [3] show the accuracy of the reconstruction improves because the encoder is able to learn more robust feature extractors. The textureless dataset described in Section 4.1 was used for training and testing this network, and experiments showed poor reconstruction accuracy for 3D meshes compared to normal and depth maps.

The network was trained using the Adam optimizer [25] with a fixed learning rate of $10^{-3}$ and κ=10. The authors used Keras [26] with a Tensorflow [27] backend for implementation and published the source code. At run-time, the network takes 0.016 s to predict both depth and normal maps together, and 0.01 s when predicting either the depth or normal map individually.

### 5.2. Patch-Net

**Figure 4:**
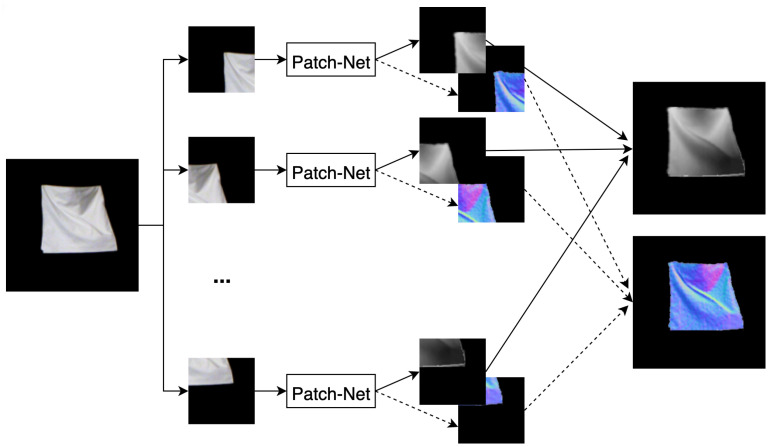
Patch-Net uses Bednarik et al. [3]’s network with only depth and normal decoders. The input image is divided into overlapping patches, and predictions for each patch are obtained separately. Patch predictions are stitched to form the complete depth and normal maps.

Tsoli and Argyros [4] proposed a patch-based variation for better textureless reconstruction. They take the network from [3] and change the block sizes to match VGG-16 [24], i.e., 64, 128, 256, 512, 512. They also remove the mesh decoder, keeping only the normal and depth decoders. They divide the input image into overlapping patches and get per patch reconstructions for normal and depth maps. These patches are then stitched together to get the final normal and depth maps at the input image resolution, and use bilateral filtering to smooth out inconsistencies that were not resolved by stitching. They call this network Patch-Net (Figure 4). Since the network expects a 224×224 spatial size of the input, each patch can have that size with the full image being even larger. This allows Patch-Net to get a higher resolution reconstruction than [3] with better accuracy and generalization. It uses the loss functions of Equations (2) and (1) on each patch to compute the normal and depth loss respectively.

The network was trained using the Adam optimizer with a fixed learning rate of $10^{-3}$. The authors extended the source code from [3], and trained their network on an Nvidia Titan V GPU with 12 GB memory. This code is not publicly available, and the authors do not report inference-time performance.

### 5.3. HDM-Net

**Figure 5:**
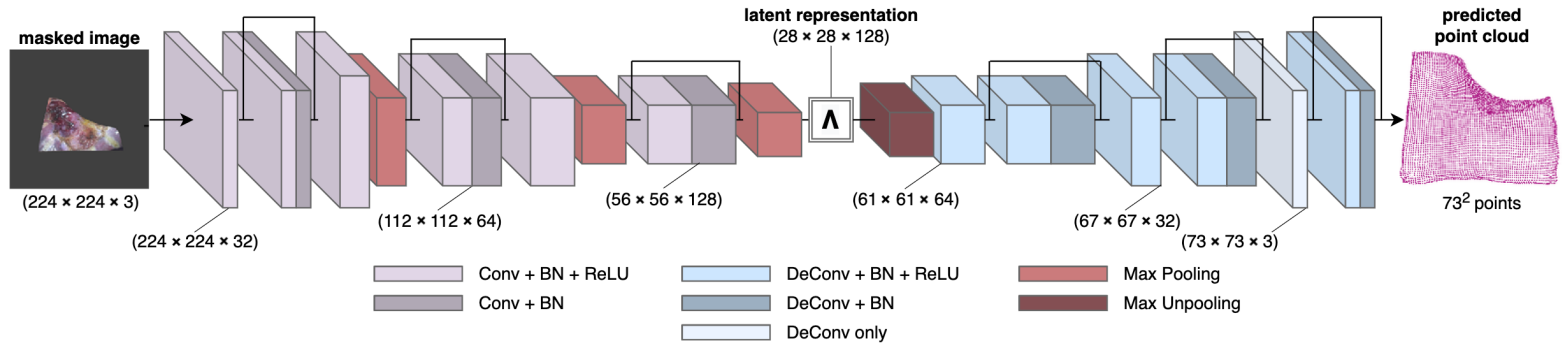
Overview of the HDM-Net [5] architecture. It has an encoder that takes an RGB image of size 224×224×3 and encodes it into a latent representation of size 28×28×128. This is then used by the decoder to reconstruct a 3D point cloud of the surface with $73^{2}$ points.

The *Hybrid Deformation Model Network* (HDM-Net) [5] is another approach for reconstructing deformable surfaces from a single-view. Like [3] and Patch-Net, HDM-Net uses an encoder–decoder architecture (Figure 5), but with only one decoder instead. However, the encoder and decoder are not symmetric to each other in this network. They also have a smaller depth, with only 9 convolution layers in the encoder instead of 13 convolution layers in the VGG-16-based architectures. The upsampling in the decoder is performed using transposed convolutions, as in [28], except at the first decoder layer where a non-linear max-unpooling operation similar to [3,4,23] is used. HDM-Net directly learns the 3D shape and gives a dense reconstruction of the surface of size 73×73×3 as a point cloud. It is trained on the synthetic point cloud data (Section 4.2) of a thin non-rigid plate undergoing various non-linear deformations, with a known shape at rest. Three different domain-specific loss functions are used to jointly optimize the output of the network, with the goal of learning texture-dependent surface deformations, shading, and contours for effective handling of occlusions.

The first loss function is a common 3D regression loss that computes the 3D error by penalizing the difference between the predicted 3D geometry $S_{n}$ and ground-truth 3D geometry $S_{n}^{\prime }$, that is,

(6) $$L_{3D}=\frac{1}{N}\sum_{n=1}^{N}{∥S_{n}^{\prime }-S_{n}∥}_{F}^{2}$$

where ${∥\cdot ∥}_{F}$ is the Frobenius norm. For each state *n*, the squared Frobenius norm of the difference between predicted and ground-truth geometries is calculated, and then averaged for all *N* states.

An isometry prior is used to constrain the regression space using an isometric loss that penalizes the roughness in the predicted surface by ensuring that neighboring vertices are located close to each other. The loss function is expressed in terms of the predicted geometry $S_{n}$ and its smooth version ${\bar{S}}_{n}$

(7) $$L_{iso.}=\frac{1}{N}\sum_{n=1}^{N}{∥{\bar{S}}_{n}-S_{n}∥}_{F}$$

with

(8) $${\bar{S}}_{n}=\frac{1}{2\pi \sigma^{2}}\exp\left(-\frac{x^{2}+y^{2}}{\sigma^{2}}\right)*S_{n}$$

where ∗ is a convolution operator and $\sigma^{2}$ is the variance of Gaussian, and *x* and *y* stand for the point coordinates.

The third loss function optimizes the contour shapes by computing a reprojection loss. The predicted and ground-truth 3D geometries are first projected onto a 2D plane and before computing their difference as

(9) $$L_{cont.}=\frac{1}{N}\sum_{n=1}^{N}{∥\tau \left(\pi \left(S_{n}\right)\right)-\tau \left(\pi \left(S_{n}^{\prime }\right)\right)∥}_{F}^{2}$$

where π is a differentiable 3D to 2D projection function and τ is a function that thresholds all positive values to 1 using a combination of tanh and ReLU. This gives contours as 0–1 transitions. The total loss is computed by adding all three losses with equal weights.

HDM-Net was trained for 95 epochs on a GEFORCE GTX 1080Ti GPU with 11 GB of global memory. The training relied on the PyTorch framework [29] and took 2 days to complete. At inference time, the network can reconstruct frames with a frequency of 200 Hz, or 0.005 s per frame. The source code was not published.

### 5.4. IsMo-GAN

**Figure 6:**
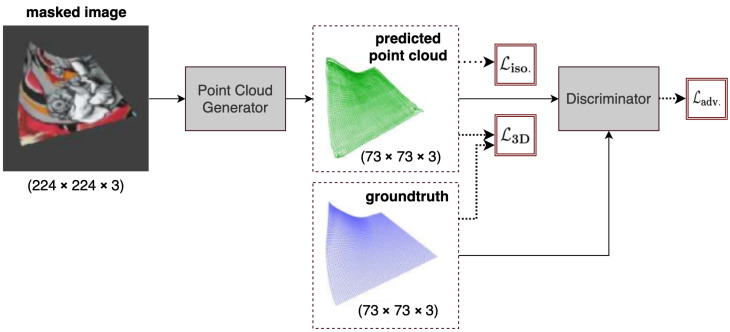
Overview of IsMo-GAN [6]. The generator network accepts a masked RGB image, segmented by the object detection network (OD-Net), and returns a 3D point cloud. The output and ground-truth are fed to the discriminator which serves as a surface regularizer.

An improved version of HDM-Net is the Isometry-Aware Monocular Generative Adversarial Network (IsMo-GAN) [6], which introduces two key modifications to achieve 10–30% reduction in reconstruction error in different cases, including reconstruction of textureless surfaces. First, IsMo-GAN has an integrated Object Detection Network (OD-Net) that generates a confidence map separating the background from the foreground. Secondly, IsMo-GAN is trained in an adversarial setting, which is different from the training of the simple auto-encoder-based networks discussed in previous sections. The OD-Net is a simplified version of U-Net [28] with fewer layers than the original. It takes a 224×224×3 RGB image and outputs a grayscale confidence map indicating the position of the foreground in the image. The confidence map is binarized [30] and the target image is extracted using Suzuki et al.’s algorithm [31]. The masked-out input image is then passed to the Reconstruction Network (Rec-Net), which has skip connections like HDM-Net and has a similar architecture but with fewer layers. Like HDM-Net, the Rec-Net outputs a 73×73×3 size point cloud. OD-Net and Rec-Net together make up the generator of IsMo-GAN. The discriminator network consists of four convolution layers followed by a fully connected layer and a sigmoid function. IsMo-GAN uses the LeakyReLU activation everywhere, instead of ReLU which was used in all other networks discussed previously. Figure 6 shows an overview of the IsMo-GAN network.

IsMo-GAN penalizes the output of the Rec-Net with the 3D loss (Equation (6)) and isometric loss (Equation (7)) from HDM-Net, where the predicted geometry is equal to the generator output on the input image, i.e., $S_{n}=G\left(I\right)$. In addition to this, for adversarial training, IsMo-GAN uses cross entropy (BCE) [32], defined as

(10) $$L_{G}=-\frac{1}{MN}\sum_{m=1}^{M}\sum_{n=1}^{N}\log\left(D(G\left(I_{m}^{n}\right))\right)$$

for the generator G, and

(11) $$L_{D}=-\frac{1}{MN}\sum_{m=1}^{M}\sum_{n=1}^{N}\left[\log\left(D\left(S_{m}^{\prime }\right)\right)+\log\left(1-D\left(G\left(I_{m}^{n}\right)\right)\right)\right]$$

for the discriminator D, where *M* is the number of states, and *N* is the number of images for each state. The adversarial loss is then defined as the sum of the generator and discriminator losses

(12) $$L_{adv}=L_{G}+L_{D},$$

and it represents the overall objective of the training which encourages IsMo-GAN to generate more realistic surfaces. It is a key component that lets IsMo-GAN outperform HDM-Net [5] by 10–15% quantitatively as well as qualitatively on real images. The adversarial loss makes up for the undesired effects of the 3D loss and the isometry prior by acting as a novel regularizer for the surface deformations. This network is trained and evaluated on the same dataset as HDM-Net, as well as on the 3D mesh data of the cloth object from the subset of the Bednarik et al. [3]’s real textureless surfaces dataset.

The OD-Net and Rec-Net were both trained separately for 30 and 130 epochs respectively, using the Adam optimizer with a fixed learning rate of $10^{-3}$ and a batch size of 8. IsMo-GAN was implemented using PyTorch, but the source code was not made public. It takes 0.004 s to run an inference, which is a 20% improvement over HDM-Net.

### 5.5. Pixel2Mesh

**Figure 7:**
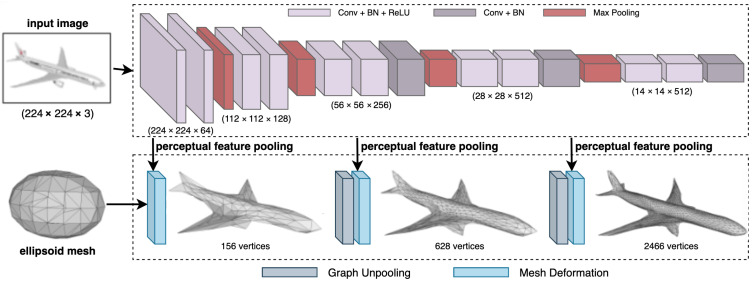
The Pixel2Mesh [7] network consists of two parallel networks that take an RGB image and a coarse ellipsoid 3D mesh, and learn to regress the 3D shape of the object in the image. The key contribution is the graph-based convolutions and unpooling operators in the bottom half of the network.

Pixel2Mesh [7] is a deep learning network that reconstructs 3D shape as a triangulated mesh from a single RGB image. It was proposed in 2018 [7] and is one of the earliest methods for monocular 3D reconstruction. Its primary idea is to use graph-based convolutions [33] to regress the mesh vertices. The network is made up of two parts: a VGG-16-based feature extractor and a graph-based convolution network (GCN). The feature extractor network takes a 224×224 image to reconstruct. Additionally, the GCN takes an ellipsoid mesh with 156 vertices and 462 edges. The feature extractor network then feeds the extracted perceptual features at different stages to the GCN in a cascaded manner, which refines the initial mesh in a coarse-to-fine manner by adding details at each stage. The GCN finally outputs a mesh with 2466 vertices (Figure 7). Each mesh deformation block in the GCN is made of 14 layers of graph-based convolutions with ResNet-like [34] skip connections. Their job is to optimize the position of existing vertices to get a mesh matching the object shape. This is followed by a graph unpooling layer that interpolates the mesh to increase the number of vertices.

Pixel2Mesh combines four different loss functions to optimize its weights. These include the Chamfer loss [35] to constraint the location of mesh vertices, a normal consistency loss, a Laplacian regularization to maintain the neighborhood relationships when deforming the mesh, and an edge length loss to prevent outliers. The total loss is then calculated as a weighted sum of the individual losses. The network is trained and evaluated on the R2N2 subset [18] of the ShapeNet dataset [10], which consists of synthetically rendered images and 3D mesh ground truth. The network is also qualitatively evaluated on the Stanford Online Products dataset [36], which contains real-world images of objects without any 3D labels.

Pixel2Mesh was implemented using Tensorflow and the official source code is available on GitHub. It used the Adam optimizer with a weight decay of $1^{-5}$ and a batch size of 1 to train for 50 epochs, with the initial learning rate of $3^{-5}$. The training took 72 h on Nvidia Titan X GPU with 12 GB memory, and the trained network can reconstruct a mesh containing 2466 vertices in 15.58 ms.

### 5.6. Salvi et al.

A new category of networks is adding self-attention modules [37,38] to 3D reconstruction networks. Salvi et al. [8] proposed one such network, which improves Occupancy Networks (ONets) [39] by adding self-attention to them. ONets consist of three parts: a feature extractor, a decoder, and a continuous decision boundary function, called the occupancy function o:R→{0,1}, that classifies each point from the space as whether or not it belongs to the surface. This provides a general 3D representation that allows extracting meshes at any resolution. ONets are an extension of the autoencoders discussed in previous sections, where the encoders functioned as feature extractors, followed by a decoder to reconstruct the 3D shape.

**Figure 8:**
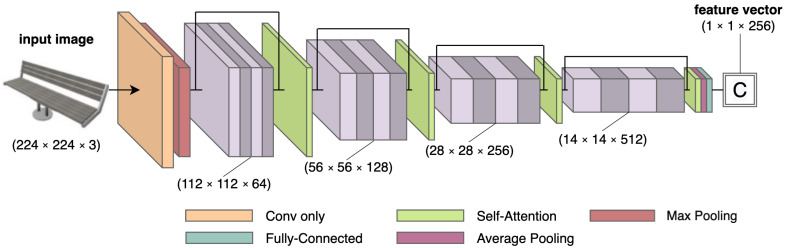
The “attentioned” ResNet-18 [34] network with four self-attention blocks [37] added to it. This encoder network is used by [8] to extract image features, which are fed to a decoder with five Conditional Batch Normalization blocks followed by an occupancy function.

In the networks discussed previously as well as ONets introduced in [39], the feature extractors are based on CNNs. Standard CNNs work with local receptive fields and need very deep architectures to successfully model global dependencies. This is because the features they learn are relatively shallow and do not capture the long-range correlations in natural images. To address this limitation, self-attention modules were introduced that calculate the response at a given position as a weighted sum of the features at all positions. This allows them to efficiently model global dependencies with much smaller networks than traditional CNNs. Salvi et al. [8] show that adding self-attention modules at different locations in the feature extractor can improve the performance of an Occupancy Network. When used earlier in the network, self-attention allows the network to focus more on finer details. When used later in the network, it allows the network to extract better structural features. Figure 8 depicts one such feature extractor proposed by [8], showing a ResNet-18 [34] network with four self-attention modules.

They train their network on the synthetic R2N2 dataset [18] (see Section 4.4) using an ensemble approach, where the ensemble is made up of one specialized ONet for each object type. This is supported by their experiments which show that self-attention-based ONets have better results if trained for each category separately. The network was also qualitatively evaluated on a subset of the Stanford Online Products dataset [36], which contains real images, and showed a more consistent and better reconstruction of meshes when compared to existing approaches. Self-attention in decoders was not used due to computational limitations.

Adam optimizer with a learning rate of $10^{-3}$ and weight decay of $1^{-5}$ was used for training the network for 200 K steps. All other hyperparameters were kept the same as in [39]. The source code for this network is not available.

### 5.7. VANet

**Figure 9:**
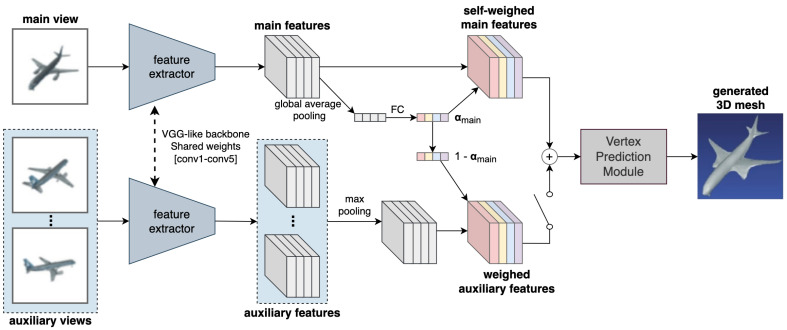
Overview of VANet [9], a unified approach for both single and multi-view reconstruction with a two-branch architecture.

Another network that uses the attention mechanism is the View Attention Guided Network (VANet) [9]. It uses channel-wise view attention and a dual pathway network for better reconstruction of occluded parts of the objects, and defines a unified approach for both single and multi-view reconstruction. As shown in Figure 9, the proposed architecture consists of a main pathway and an auxiliary pathway. The main path uses the first view of a scene to reconstruct a 3D mesh. If any more views are available, they are then fed to the auxiliary path, which aligns them with the main view and uses the additional information from these new views to refine the reconstructed mesh. The main view features after the encoder are pooled along the spatial dimensions using global average pooling to get a channel descriptor of shape 1×1×C. This is then sent to a system of fully connected layers followed by a sigmoid function to generate a channel-wise attention map $\alpha_{main}$. These attention weights are then used to re-calibrate the computed feature maps. If auxiliary views are available, they are used to enhance the less-visible parts in the original view. A max pooling operation is used to select permutation invariant auxiliary view features, which are multiplied by $1-\alpha_{main}$ and finally added to the main view features. These are then sent to a vertex prediction module to generate the reconstructed 3D mesh. The vertex prediction module is based on the mesh deformation module of Pixel2Mesh [7].

VANet is trained using the same four loss functions as Pixel2Mesh, and evaluated on the R2N2 subset [18] of the ShapeNet dataset [10]. Using the Adam optimizer with an initial learning rate of $2\;\times \;10^{-5}$ and a batch size of 1, the network was trained for 20 epochs. It was implemented in Tensorflow but the source code was not published.

### 5.8. 3D-VRVT

**Figure 10:**
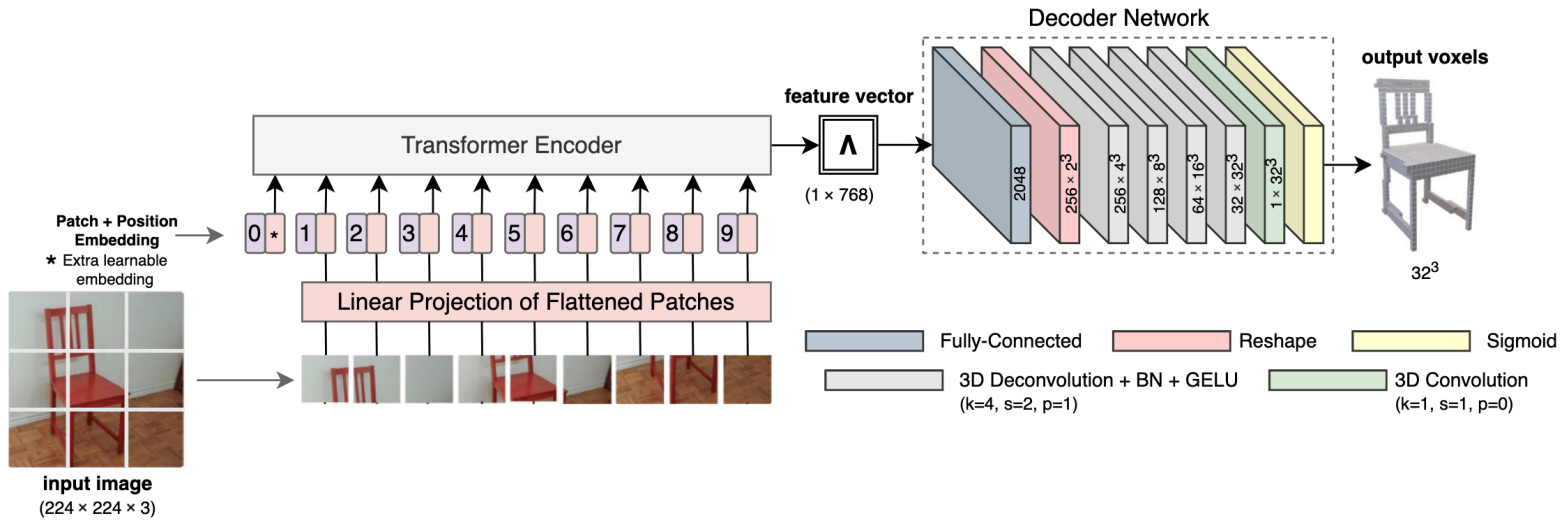
3D-VRVT takes one image as input and uses a Vision Transformer encoder to extract a feature vector. This is then fed to a decoder that outputs the voxel representation of the object.

Vaswani et al. [37] initially proposed the Transformer architecture for natural language processing (NLP) tasks. These methods used the self-attention mechanism to let the network understand longer sequences of text to compute a representation for the whole sequence. Salvi et al. [8] used the self-attention mechanism from Transformers in their “attentioned” ResNet encoder to extract better features for 3D reconstruction. However, their input is not sequential (Section 5.6). Dosovitskiy et al. [40] proposed a novel architecture called Vision Transformers that breaks down images into patches and treats those patches as part of a sequence. Using a linear projection, vector embeddings for each patch are obtained. This sequence of patch embeddings is then fed to a Transformer network. Inspired by this, Li and Kuang [41] proposed a Vision Transformer-based network (Figure 10) for reconstructing voxels from a single image. They call this network 3D-VRVT.

3D-VRVT uses a Vision Transformer as an encoder, that takes a 224×224 RGB image as input and produces a feature vector of size 768 which is fed to a decoder network. The decoder network has a fully connected layer that upscales the feature vector to 2048 and then reshapes it into a 3D tensor of shape $256\times 2^{3}$. This is followed by four 3D deconvolutions with a kernel size of 4, stride 2, and padding 1 that iteratively refine the 3D grid until it has the resolution $32\times 32^{3}$. Each deconvolution operation is also followed by a 3D batch normalization and a GELU activation function. Then, a final deconvolution with kernel size 1 is applied to get a grid of $1\times 32^{3}$. This is passed through a sigmoid activation function before getting the final voxel output.

**Table 3:** Summary of all 3D reconstruction networks discussed in this paper.

Literature	Architecture	Output	Method	Dataset Type	Runtime [s]
Bednarik et al. [3] (2018)	VAE with one encoder and three decoders	normal map, depth map, and 3D mesh	based on SegNet [23] with VGG-16 [24] backbone	real, deformable, textureless surfaces	0.016
Patch-Net [4] (2019)	VAE with one encoder and two decoders	normal and depth maps	converts image to patches, gets 3D shape of patches using [3], and stitches them together	real, deformable, textureless surfaces	-
Hybrid Deformation Model Network (HDM-Net) [5] (2018)	VAE with one encoder and one decoder	3D point cloud	simple autoencoder with skip connections like ResNet [34], combines 3D regression loss with an isometry prior and a contour loss	synthetic, deformable, well-textured surfaces	0.005
Isometry-Aware Monocular Generative Adversarial Network (IsMo-GAN) [6] (2019)	GAN with two sequential VAEs as a generator and a simple CNN as discriminator	3D point cloud	integrates an OD-Net to segment foreground, and trains in an adversarial setting along with 3D loss and isometry prior from [5]	synthetic, deformable, well-textured surfaces and real, deformable, textureless surfaces	0.004
Pixel2Mesh [7] (2018)	two-lane network with a feature extractor and a graph based mesh predictor (GCN)	3D mesh	feature extractor based on VGG-16 [24], feeds cascaded features to the GCN that uses graph convolutions	synthetic, rigid, well-textured surfaces	0.016
View Attention Guided Network (VANet) [9] (2021)	two-lane feature extractor for both single or multi-view reconstruction, followed by a mesh prediction network	3D mesh	uses channel-wise attention and information from all available views to extract features, which are then sent to a Pixel2Mesh-based [7] mesh vertex predictor	synthetic, rigid, well-textured surfaces	-
Salvi et al. [8] (2020)	VAE based on ONets [39] with self-attention [37] in encoder	parametric representation	ResNet-18 [34] encoder with self-attention modules, followed by a decoder and an occupancy function	synthetic, rigid, well-textured surfaces	-
3D-VRVT [41] (2021)	encoder-decoder architecture	voxel grid	encoder based on Vision Transformers [40], followed by a decoder made up of 3D deconvolutions	both synthetic and real, rigid, well-textured surfaces	0.009

The network was trained on the ShapeNet dataset. It used an SGD optimizer and a warm-up cosine annealing learning rate with a momentum of 0.9. The learning rate ranged between $2^{-5}$ and $2^{-3}$. The training relied on a PyTorch implementation and continued for 600 epochs on Nvidia Titan V GPU, including 10 warm-up epochs. At test time, it takes 8.82 ms to reconstruct an object with this network.

## 6. Comparison

We described different 3D reconstruction methods in this paper, which were trained by their authors on various datasets and evaluated on different error metrics. In this section, we first formally define the various error metrics used for the evaluation of 3D reconstruction methods. We then describe comparable experiments on similar datasets by different networks, and report and compare the performance of those methods.

### 6.1. Metrics

1. *Depth Error ($E_{D}$)*: The depth error metric is used to compute the accuracy of depth map predictions. Let $\Theta_{K}$ and $\Theta_{K}^{\prime }$ be the point clouds associated with the predicted and ground-truth depth maps respectively, with the camera matrix K. To remove the inherent global scale ambiguity [42] in the prediction, $\Theta_{K}$ is aligned to ground-truth depth map $D^{\prime }$ to get an aligned point cloud ${\bar{\Theta }}_{K}$ as
  (13) $${\bar{\Theta }}_{K}=\Omega \left(\Theta_{K},D^{\prime }\right)$$
  where Ω is the Procrustes transformation [43]. Then, the depth error $E_{D}$ is calculated as
  (14) $$E_{D}=\frac{1}{N}\sum_{n=1}^{N}\frac{\sum_{i}∥\Theta_{K}^{\prime }-{\bar{\Theta }}_{K}∥B_{i}^{n}}{\sum_{i}B_{i}^{n}}.$$
  Note that the foreground mask B in the equation ensures that the error is only calculated for foreground pixels. Smaller depth errors are preferred.
2. *Mean Angular Error ($E_{MAE}$)*: The mean angular error $E_{MAE}$ metric is used to calculate the accuracy of normal maps, by computing the average difference between the predicted and ground-truth normal vectors. The angular errors for all samples are calculated using Equation (3), and then averaged for all samples. Smaller angular errors indicate better predictions.
3. *Volumetric IoU ($E_{IOU}$)*: The Intersection over Union (IoU) metric for meshes is calculated as the volume of the intersection of ground-truth and predicted meshes, divided by the volume of their union. Larger values are better.
4. *Chamfer Distance ($E_{CD}$)*: Chamfer distance is a measure of similarity between two point clouds. It takes the distance of each point into account by finding, for each point in a point cloud, the nearest point in the other cloud, and summing their squared distances.
  (15) $$E_{CD}=\frac{1}{\left|\Theta \right|}\sum_{x\in \Theta }\min_{y\in \Theta^{\prime }}{∥x-y∥}^{2}+\frac{1}{|\Theta^{\prime }|}\sum_{x\in \Theta^{\prime }}\min_{y\in \Theta }{∥x-y∥}^{2}$$
  where ${∥\cdot ∥}_{2}$ is the square of Euclidean distance. A smaller CD score indicates a better value.
5. *Chamfer-L1 ($E_{CD_{1}}$)*: The Chamfer distance (CD) has a high computational cost for meshes because of a large number of points, so an approximation called Chamfer-L1 is defined. It uses the L1-norm instead of the Euclidean distance [8]. Smaller values are preferred.
6. *Normal Consistency ($E_{NC}$)*: The normal consistency score is defined as the average absolute dot product of normals in one mesh and normals at the corresponding nearest neighbors in the other mesh. It is computed similarly to Chamfer-L1 but the L1-norm is replaced with the dot product of the normal vectors on one mesh with their projection on the other mesh [8]. Normal consistency shows how similar the shapes of two volumes are, and is useful in cases such as where two meshes might overlap significantly, giving a high IoU, but have a different surface shape. Higher normal consistency is preferred.
7. *Earth Mover’s Distance ($E_{EMD})$*:
  The Earth Mover’s Distance computes the cost of transforming one one pile of dirt, or one probability distribution, into another. It was introduced in [44] as a metric for image retrieval. In case of 3D reconstruction, it computes the cost of transforming the set of predicted vertices into the ground-truth vertices. The lower the cost, the better the prediction.
8. *F-score ($E_{F}$)*:
  The F-score evaluates the distance between object surfaces [2,45]. It is defined as the harmonic mean between precision and recall. Precision measures reconstruction accuracy by counting the percentage of predicted points that lie within a certain distance from the ground truth. Recall measures completeness by counting the percentage of points on the ground truth that lie within a certain distance from the prediction. The distance threshold τ can be varied to control the strictness of the F-score. In the results reported in this paper, $\tau =10^{-4}$.

### 6.2. Experiments

The RGB-D dataset consisting of normal and depth maps of real data [3] was used by Bednarik et al. and Patch-Net in their experiments. This dataset contains five different textureless surfaces. Experiments were conducted where the network was trained with samples from one surface and then evaluated on samples from another surface. For example, in the experiment “cloth-hoody”, the network was trained on the cloth object and evaluated on the hoody object. The depth and angular errors for these experiments are summarized in Table 4. Patch-Net outperforms [3] in almost all experiments on both the metrics.

The HDM-Net and IsMo-GAN networks were evaluated on the synthetically generated point cloud dataset consisting of a thin, deforming plate with various textures and under different illuminations [5]. The 3D error $E_{3D}$ and its standard deviation over a set of frames $E_{\sigma }$ are reported in Table 5. Results are reported for each of the four textures in the network, under a constant illumination.

For a small subset of the cloth object in the textureless dataset, ground-truth 3D meshes are also provided. For this cloth data, the 3D error is reported for [3], HDM-Net, and IsMo-GAN. In addition, the authors of [6] removed the textures from surfaces in the synthetic thin plate data to create another textureless data. They report the 3D error on this data for HDM-Net and IsMo-GAN. We summarize these results for textureless mesh and point cloud reconstruction in Table 6.

For most other experiments, the Choy et al. [18] subset of the ShapeNet dataset [10], described in Section 4.4, is used. For each of the 13 objects in this dataset, various error metrics are reported for the task of mesh reconstruction. Table 7 summarizes these results, reporting the F-score, Earth Mover’s Distance, and the Chamfer Distance metrics. Salvi et al. [8] report the IoU, Chamfer-L1, and normal consistency metrics on the same dataset. These results are summarized in Table 8.

## 7. Discussion

There are many different methods for 3D reconstruction from 2D images. In this paper, we discussed several of them—including those that use depth maps, normal maps, point clouds, surface meshes, or volumetric data. Each of these approaches has its own advantages and disadvantages, but no one method is perfect. This makes the task of 3D reconstruction an ill-posed problem, which requires careful consideration when choosing a method to use. In single-view 3D reconstruction, this becomes even more challenging because the network has to reconstruct the shape of surfaces that may not even be visible in the image at all.

We discussed two methods that reconstruct the 3D shape of textureless surfaces. In general, the more distinctive textures an image contains, the easier it is to reconstruct in 3D. With only a single RGB image—that contains surfaces with no distinctive textures—dense reconstruction in 3D can be very difficult. That is why Bednarik et al. [3] and Patch-Net only reconstruct the so-called 2.5D shape in form of normal and depth maps. They both use a very similar network architecture, but the authors of [4] use a patch-based strategy instead of reconstructing the whole image together. By doing so, they are able to reduce the depth error by 25.3% and the mean angular error of the surface normals by 13.0%. Later, Shimada et al. [6] used a subset of the same textureless surfaces data to directly reconstruct 3D meshes. They report results for HDM-Net and their own IsMo-GAN network, showing 17.8% and 26.5% improvement respectively over [3]’s original textureless surfaces method for meshes.

HDM-Net, like [3], is an encoder-decoder network and has a similar VGG-16 backbone but fewer convolutional layers. The major difference in the encoder is the ResNet-like skip connections. In [3], the decoder consisted of only one convolutional layer, followed by pooling and a fully connected layer. HDM-Net uses a much larger decoder with transposed convolutions for upsampling. Finally, while [3] for its mesh decoder used a simple mean squared error loss function, HDM-Net combines that with two more loss functions that enforce isometry and contour constraints. The skip connections, a larger decoder, and domain-specific loss functions help HDM-Net get a significantly improved reconstruction compared to [3]. IsMo-GAN takes the isometry constraints from HDM-Net and uses an adversarial training setting to improve the mesh reconstruction accuracy for textureless surfaces even further.

HDM-Net and IsMo-GAN also evaluate their methods on a synthetic dataset of a deforming plate with a variety of textures. In addition to the adversarial training, IsMo-GAN’s integrated Object Detection Network and the choice of LeakyReLU activation instead of ReLU help it outperform HDM-Net when reconstructing 3D point clouds by 10–30% in various experiments.

Next, we talked about two methods that reconstruct 3D meshes using Choy et al. [18]’s rendered images of the ShapeNet dataset; Pixel2Mesh [7] and VANet. Both use a dual-lane architecture with separate roles for each lane. Pixel2Mesh has one lane for feature extraction, which feeds cascaded input to the other lane that performs the reconstruction. On the other hand, VANet’s two lanes are both for feature extraction, and have shared weights. VANet can be used for both single and multi-view reconstruction, and the first lane extracts features from the main view while the second from all auxiliary views. The feature vectors extracted from both lanes are concatenated and sent to another module that has an architecture similar to Pixel2Mesh’s mesh reconstruction lane. This uses graph-based convolutions to generate a mesh from extracted features. By primarily improving their feature extractor, which uses channel-wise attention, VANet was then able to use the same reconstruction network as Pixel2Mesh to get up to 40% reduction of the average Chamfer distance error (see Table 7).

Finally, we discussed Salvi et al. [8]’s attentioned occupancy network for reconstructing the 3D shape as a continuous function. The motivation for this type of output is to have a standard method for efficiently representing a 3D shape. In this network, an encoder with ResNet-18 backbone is augmented with several self-attention blocks to improve comprehension of global dependencies in the input images. This is then passed to a decoder network and an occupancy function to get the final output, which can then be used to obtain a mesh at any resolution. The mesh output is compared to Pixel2Mesh using several metrics, and Salvi et al. [8] get 11 times smaller average Chamfer-L1 distance than Pixel2Mesh. No metrics directly comparing this method with VANet are reported, but as this also uses the same ShapeNet dataset, and the reported Chamfer-L1 distance is a variant of the standard Chamfer distance reported by VANet, it can be assumed that self-attention in VANet’s encoder would improve reconstruction accuracy of that network, as well. However, as the authors of [8] showed in some of their experiments, the benefit from self-attention modules was highest when used at the early layers of the encoder as opposed to the later layers.

## 8. Conclusions

This paper reviewed several methods for monocular 3D reconstruction and concluded that it remains an ill-posed problem. This is primarily because of a lack of a standardized, one-size-fits-all method of representing 3D shapes, as well as the non-availability of standard datasets that cover the whole range of objects to be reconstructed. For example, while there are a lot of large-scale synthetic datasets, real-world 3D datasets are rare and smaller on average. This is because of the inherent difficulty of capturing real 3D data. When it comes to textureless surfaces, there are even fewer datasets available, and those that are available only contain RGB-D data. Standard datasets for deformable textureless surfaces with ground-truth point clouds or meshes provided are not available. This is why most textureless reconstruction methods only reconstruct depth maps or surface normals.

As discussed before, most networks in 3D reconstruction are made up of encoder-decoder architectures, which in turn are based on semantic segmentation networks such as SegNet and UNet. Various enhancements in these networks have shown promise, including the use of domain-specific losses that enforce various shape constraints on the surfaces. An example of this is the isometry prior used by some networks. Other networks have shown that using ResNet-like skip connections improves the representation power of the network, giving better results. Attention-based methods have also shown good results, with self-attention modules when compared with ResNet’s skip connections, improving the performance of previous methods manifolds. However, self-attention is an expensive process that cannot be practically used everywhere in the encoder–decoder networks. Instead, their use is limited to the earlier layers of the encoder. Like self-attention, the use of max pooling indices from the encoder layers in the decoder layers is also an expensive process as it requires additional memory at each forward pass. Some networks have successfully used interpolation and transposed convolutions for upsampling, eliminating the need for pooling indices from the encoder. Using adversarial training in a GAN-based network also showed significantly better results. The GAN-based network also performed significantly faster than all other methods reviewed in this paper, generating point clouds at 250 Hz frequency, thus making it suitable for runtime 3D reconstruction. Transformers are also gaining traction for vision tasks, with one network using Vision Transformers for single-view 3D reconstruction. This network shows promising results in reconstructing a low-resolution voxel grid, and improves the IoU on the ShapeNet dataset by 9% compared to other state-of-the-art methods.

In the future, 3D reconstruction research can benefit from more datasets, especially for textureless surfaces and from real-world objects. For depth maps and surface normals, simple RGB-D cameras such as Microsoft Kinect may be used, but it is generally very challenging to collect real-world datasets of the same size as can be generated synthetically. Collecting ground-truth point clouds or 3D meshes from real-world objects poses an even bigger challenge because of the more specialized equipment required. Synthetic datasets focusing on textureless surfaces, in particular, and including both deforming and rigid surfaces, can be generated using software like Blender. In addition to dataset creation, methods to deal with the bottlenecks created by self-attention modules and pooling indices also need to be studied. The full potential of Transformers in the context of single-view 3D reconstruction is also still unknown. Finally, the use of adversarial training and generator-discriminator networks for 3D reconstruction needs to be further explored.

## Figures and Tables

**Table 1:** Summary of the major 3D datasets in this paper. Most of the datasets contain textured surfaces and are generated from synthetic 3D models. Real datasets captured directly with 3D sensors are less common and smaller in size because of the difficulty associated with obtaining them.

Dataset	Type	Surface	Texture	Groundtruth	Size
Bednarik et al. [3]	Real	Deformable	No	Depth and Normal Maps	26,445
Golyanik et al. [5]	Synthetic	Deformable	Yes	Point Clouds	4648
ShapeNet [10]	Synthetic	Mixed	Yes	3D Mesh	>300 M
R2N2 [18]	Synthetic	Rigid	Yes	3D Meshes and Voxelized Models	50,000

**Table 2:** Summary of objects in the textureless surfaces dataset [3]. Sequences of data samples were captured using a Kinect device at 5 FPS with varying lighting conditions across sequences.

	cloth	tshirt	sweater	hoody	paper
sequences	18	12	4	1	3
samples	15,799	6739	2203	517	1187

**Table 4:** The textureless surfaces dataset [3] is used to compare the performance of different depth and normal map reconstruction methods. 128×128 size patches were used in the Patch-Net. Bold values show the best results for each metric.

Metric	$E_{D}$ (mm) ↓		$E_{\mathrm{MAE}}$ (degrees) ↓	
**Method**	**Bednarik et al. [3]**	**Patch-Net [4]**	**Bednarik et al. [3]**	**Patch-Net [4]**
cloth-cloth	17.53±5.50	12.80±4.45	17.37±12.51	14.72±3.39
tshirt-tshirt	17.18±18.58	13.70±3.83	18.07±12.71	18.63±4.43
cloth-tshirt	26.26±7.72	22.74±7.20	25.74±15.81	24.29±3.80
cloth-sweater	38.93±10.36	30.10±10.00	31.52±19.07	27.94±4.79
cloth-hoody	43.22±24.81	31.09±8.73	32.54±21.15	29.73±2.52
cloth-paper	24.16±7.15	14.53±4.48	35.53±22.16	24.52±5.96

**Table 5:** Comparison of different point cloud reconstruction methods using the synthetic thin plate dataset [5] under one illumination. Bold values show the best results for each metric.

Metric	$E_{3D}$ (mm) ↓		$E_{\sigma }$ (mm) ↓	
**Method**	**HDM-Net [5]**	**IsMo-GAN [6]**	**HDM-Net [5]**	**IsMo-GAN [6]**
endoscopy	48.50	**33.60**	**13.50**	14.80
graffiti	49.90	**33.30**	22.00	**20.80**
clothes	48.90	**35.30**	26.40	**24.20**
carpet	144.20	**110.50**	26.90	**26.80**
mean	72.88	**53.18**	22.20	**21.65**

**Table 6:** Comparison of mesh reconstruction from textureless data. As can be seen, IsMo-GAN outperforms [3] and HDM-Net on the real textureless cloth data by 26.5% and 10.5% respectively, and it outperforms HDM-Net on the synthetic textureless thin-plate data by 31.9%. Bold values show the best results.

Metric	$E_{3D}$ (mm) ↓		
**Method**	**Bednarik et al. [3]**	**HDM-Net** [5]	**IsMo-GAN** [6]
cloth [3]	21.48	17.65	**15.79**
plate [5,6]	-	99.40	**67.70**

**Table 7:** Comparison of (**A**) Pixel2Mesh [7] and (**B**) VANet [9] on the Choy et al. [18] subset of the ShapeNet dataset [10]. VANet outperforms Pixel2Mesh in all metrics, with 10–40% improvement. Bold values show the best results for each metric.

Metric	$E_{F}$ (%) ↑		$E_{\mathrm{EMD}}$↓		$E_{\mathrm{CD}}$↓	
**Method**	**A**	**B**	**A**	**B**	**A**	**B**
plane	71.12	**77.01**	0.579	**0.486**	0.477	**0.304**
bench	57.57	**67.69**	0.965	**0.770**	0.624	**0.362**
cabinet	60.39	**63.30**	2.563	**1.575**	0.381	**0.327**
car	67.86	**69.53**	1.297	**1.185**	0.268	**0.235**
chair	54.38	**60.74**	1.399	**0.957**	0.610	**0.443**
monitor	51.39	**60.35**	1.536	**1.269**	0.755	**0.459**
lamp	48.15	**56.26**	1.314	**1.086**	1.295	**0.879**
**Method**	**A**	**B**	**A**	**B**	**A**	**B**
speaker	48.84	**53.49**	2.951	**2.283**	0.739	**0.562**
firearm	73.20	**77.24**	0.667	**0.473**	0.453	**0.333**
sofa	51.90	**56.83**	1.642	**1.376**	0.490	**0.400**
table	66.30	**70.78**	1.480	**1.173**	0.498	**0.334**
phone	70.24	**72.27**	0.724	**0.573**	0.421	**0.298**
watercraft	55.12	**62.12**	0.814	**0.718**	0.670	**0.450**
mean	59.73	**65.20**	1.380	**1.071**	0.591	**0.414**

**Table 8:** Comparison of (**A**) Pixel2Mesh [7], (**C**) Salvi et al. [8] and 3D-VRVT [41] on the Choy et al. [18] subset of the ShapeNet dataset [10]. With self-attention after ResNet layers, Ref. [8] improves IoU by 25%, normal consistency by 8.9%, and reduces the average Chamfer-L1 distance approximately 11 times. Ref. [41] further improves the IoU by 9% with a Vision Transformer architecture containing multi-headed self-attention. Bold values show the best results for each metric.

Metric	$E_{\mathrm{IoU}}$↑			$E_{{\mathrm{CD}}_{1}}$↓		$E_{\mathrm{NC}}$↑	
**Method**	**A**	**C**	**D**	**A**	**C**	**A**	**C**
plane	0.420	**0.645**	0.608	0.187	**0.011**	0.759	**0.868**
bench	0.323	0.493	**0.563**	0.201	**0.016**	0.732	**0.813**
cabinet	0.664	0.737	**0.794**	0.196	**0.016**	0.834	**0.876**
car	0.552	0.761	**0.855**	0.180	**0.014**	0.756	**0.855**
chair	0.396	0.534	**0.553**	0.265	**0.021**	0.746	**0.829**
monitor	0.490	0.520	**0.555**	0.239	**0.026**	0.830	**0.863**
lamp	0.323	0.379	**0.436**	0.308	**0.045**	0.666	**0.722**
speaker	0.599	0.660	**0.725**	0.285	**0.028**	0.782	**0.839**
firearm	0.402	0.527	**0.597**	0.164	**0.012**	0.718	**0.804**
sofa	0.613	0.689	**0.716**	0.212	**0.019**	0.820	**0.866**
table	0.395	0.535	**0.617**	0.218	**0.019**	0.784	**0.861**
phone	0.661	0.754	**0.805**	0.149	**0.012**	0.907	**0.937**
watercraft	0.397	0.568	**0.604**	0.212	**0.018**	0.699	**0.801**
mean	0.480	0.600	**0.654**	0.216	**0.019**	0.772	**0.841**

## Data Availability

Not applicable.

## Abbreviations

The following abbreviations are used in this manuscript:

2D	Two-Dimensional
3D	Three-Dimensional
CAD	Computer-Aided Design
CNN	Convolutional Neural Network
GAN	Generative Adversarial Network
NLP	Natural Language Processing
RGB	Red-Green-Blue
RGB-D	Red-Green-Blue-Depth
RNN	Recurrent Neural Network
VAE	Variational Autoencoder
VOC	Visual Object Classes
